# Herpes zoster infection increases the risk of peripheral arterial disease: A nationwide cohort study

**DOI:** 10.1097/MD.0000000000004480

**Published:** 2016-09-02

**Authors:** Te-Yu Lin, Fu-Chi Yang, Cheng-Li Lin, Chia-Hung Kao, Hsin-Yi Lo, Tse-Yen Yang

**Affiliations:** a Division of Infectious Diseases and Tropical Medicine, Department of Internal Medicine, Tri-Service General Hospital, National Defense Medical Center; b Department of Neurology, Tri-Service General Hospital, National Defense Medical Center, Taipei; c Management Office for Health Data, China Medical University Hospital; d College of Medicine, China Medical University; e Graduate Institute of Clinical Medical Science and School of Medicine, College of Medicine, China Medical University; f Department of Nuclear Medicine and PET Center, China Medical University Hospital, Taichung; g Department of Bioinformatics and Medical Engineering, Asia University, Taichung; h Graduate Institute of Chinese Medicine, China Medical University; i Molecular and Genomic Epidemiology Center, China Medical University Hospital, Taichung; j Department of Medical Laboratory Science and Biotechnology, China Medical University, Taichung, Taiwan.

**Keywords:** cohort study, peripheral arterial disease, varicella-zoster virus

## Abstract

Varicella-zoster virus infection can cause meningoencephalitis, myelitis, ocular disorders, and vasculopathy. However, no study has investigated the association between herpes zoster (HZ) and peripheral arterial disease (PAD).

We identified newly diagnosed HZ from the Taiwan's National Health Insurance Research Database recorded during 2000 to 2010, with a follow-up period extending until December 31, 2011. In addition, we included a comparison cohort that was randomly frequency-matched with the HZ cohort according to age, sex, and index year. We analyzed the risk of PAD with respect to sex, age, and comorbidities by using Cox proportional-hazards regression models.

In total, 35,391 HZ patients and 141,556 controls were enrolled in this study. The risk of PAD was 13% increased in the HZ cohort than in the comparison cohort after adjustment for age, sex, and comorbidities. The Kaplan–Meier survival curve showed that the risk of PAD was significantly higher in the HZ cohort than in the non-HZ cohort (*P* < 0.001).

This nationwide population-based cohort study revealed a higher risk of PAD in patients with HZ infection than in those without the infection. Careful follow-up and aggressive treatment is recommended for patients with HZ to reduce the risk of PAD.

## 1 Introduction

Varicella-zoster virus (VZV) causes 2 distinct clinical diseases: varicella and herpes zoster (HZ). Varicella, more commonly called chickenpox, is a primary infection resulting from exposure to the virus. The virus primarily infects children aged younger than 13 years, and the infection is characterized by the cutaneous distribution of diffuse maculopapules, vesicles, and scabs in various disease stages. VZV infections then become latent in the dorsal root ganglia and autonomic ganglia. Spontaneous reactivation or host cell-mediated immunity decrease, such as in cases of cancer, transplant, and acquired immunodeficiency syndrome, may occur later in life. Moreover, shingles is characterized by a unilateral vesicular eruption with a dermatomal distribution.^[1,2]^

Peripheral arterial disease (PAD) is a circulatory disease that impairs adequate blood flow to peripheral tissues and causes tissue damage. Atherosclerosis is the major pathophysiology of PAD. Chang et al^[3]^ observed the prevalence of PAD in the general population is 12% to 14%. The traditional risk factors for PAD are older age, male sex, hypertension, diabetes, hyperlipidemia, obesity, smoking, and a family history of vascular diseases.^[4,5]^

Varicella-zoster virus reactivation causes meningoencephalitis, myelitis, ocular disorders, and vasculopathy.^[6]^ VZV-induced vasculopathy encompasses 2 major spectrums: large and small-vessel vasculopathy. Recent epidemiological studies from Taiwan, Denmark, and the United Kingdom have revealed an increased risk of stroke after VZV infection.^[7–10]^ In addition, Wang et al^[11]^ observed that HZ infection is associated with an increased risk of acute coronary syndrome. Some studies have reported PAD after varicella infection.^[12–14]^ However, no epidemiological studies have determined the association between HZ infection and PAD. Therefore, this population-based retrospective cohort study was conducted to investigate whether HZ infection increases the risk of PAD.

## 2 Methods

### 2.1 Data source

The present study was conducted using data from the Longitudinal Health Insurance Database 2000 (LHID2000) obtained from Taiwan's National Health Insurance (NHI) program. All NHI data are collected and input into the LHID2000 by the National Health Research Institutes to provide a comprehensive record of medical care. Since 1995, the NHI program has provided health insurance coverage to 99% of the 23.75 Taiwanese residents. The representativeness of the LHID2000 to the entire Taiwan population has been validated by previous studies,^[15,16]^ and all patient information in the database are anonymized and deidentified. The diseases were coded according to the International Classification of Disease, Ninth Revision, Clinical Modification (ICD-9-CM) diagnosis codes, 2001 edition. The Ethics Review Board of China Medical University and Hospital in Taiwan approved this study (CMUH104-REC2–115).

### 2.2 Study participants

The HZ cohort comprised patients newly diagnosed with HZ (ICD-9-CM 53) between January 1, 2000 and December 31, 2010; the diagnosis date was set as the index date. Patients with a history of PAD (ICD-9-CM 440.2, 440.3, 440.8, 440.9, 443, 444.22, 444.8, 447.8, and 447.9) before the index date or incomplete age or sex information were excluded. The non-HZ cohort patients were randomly identified from the LHID2000 during the same period (2000–2010), with exclusion criteria similar to that for the HZ cohort. Four patients from the non-HZ cohort were frequency-matched with each patient from the HZ cohort with respect to sex, age (at 5-year intervals), and index year.

### 2.3 Outcome and comorbidities

The main outcome of this study was newly diagnosed PAD during follow-up. The patients were followed from the index date until PAD diagnosis, withdrawal from the insurance system, death, or December 31, 2011, whichever occurred first. We defined baseline comorbidities, namely obesity (ICD-9-CM 278), tobacco dependency (ICD-9-CM 305.1), hypertension (ICD-9-CM 401–405), hyperlipidemia (ICD-9-CM 272), heart failure (ICD-9-CM 428), coronary artery disease (CAD; ICD-9-CM 410–414), diabetes (ICD-9-CM 250), stroke (ICD-9-CM 430–438), chronic obstructive pulmonary disease (COPD; ICD-9-CM 491, 492, and 496), and asthma (ICD-9-CM 493). Acyclovir and valacyclovir were the antiviral treatment of HZ infection.

### 2.4 Statistical analysis

The distributions of demographic data and comorbidities were compared between the HZ and non-HZ cohorts by using chi-square and *t* tests to analyze categorical and continuous variables, respectively. We estimated the cumulative incidence curves of PAD for both cohorts by using the Kaplan–Meier method, and determined the curve difference of both cohorts by using the log-rank test. The incidence densities of PAD were estimated for each cohort and stratified by sex, age, and comorbidities. Univariable and multivariable Cox proportional-hazards regression analyses were performed to estimate the hazard ratios (HRs) and 95% confidence intervals (CIs) of PAD development for the HZ cohort compared with those for the non-HZ cohort. The multivariable Cox models were adjusted for age, sex, and comorbidities of obesity, tobacco dependency, hypertension, hyperlipidemia, heart failure, CAD, diabetes, stroke, COPD, and asthma. To investigate whether antiviral treatment for HZ affects the risk of PAD, we divided the HZ cohort into 2 subgroups according to the administered antiviral treatment and compared the differences in the risk of PAD. All statistical analyses were performed using SAS 9.4 software (SAS Institute, Cary, NC), and the incidence curve was calculated using R software (R Foundation for Statistical Computing, Vienna, Austria). A 2-sided *P* value of <0.05 was considered significant.

## 3 Results

**Figure 1:**
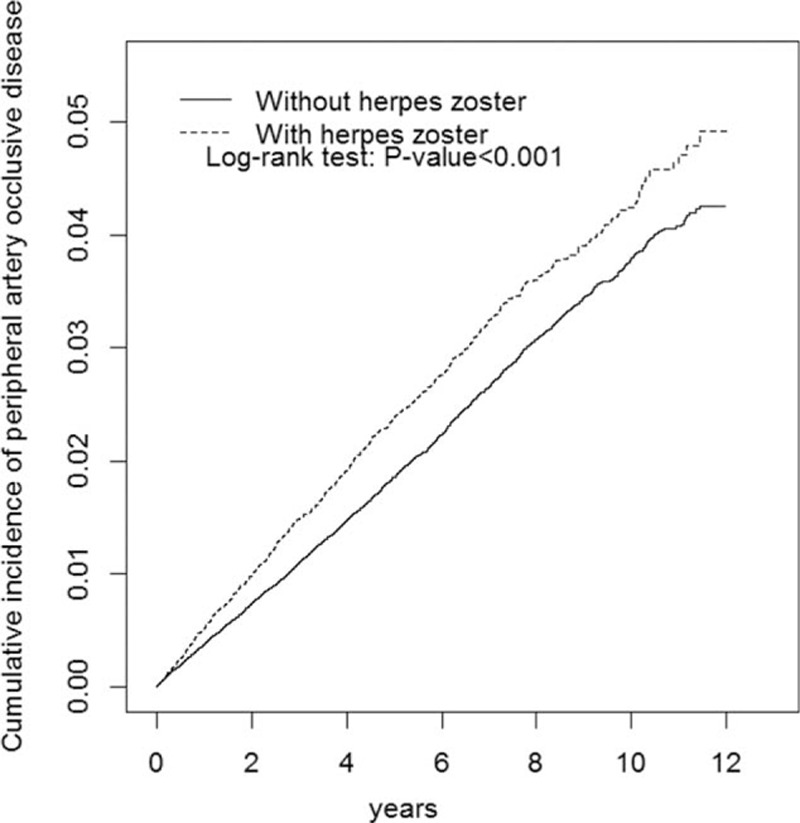
Cumulative incidence of peripheral arterial disease for patients with (dashed line) and without (solid line) herpes zoster infection.

We enrolled 35,391 patients in the HZ cohort and 141,556 patients in the non-HZ cohort, with similar sex and age distributions (Table 1). The mean age of patients in the HZ and non-HZ cohorts was 55.1 and 54.6 years, respectively, and nearly 53.2% of the patients were women. Baseline comorbidities of obesity, hypertension, hyperlipidemia, heart failure, CAD, diabetes, COPD, and asthma were more prevalent in the HZ cohort than in the non-HZ cohort (*P* < 0.05). The mean follow-up period of PAD was 4.80 (SD = 3.34) and 4.81 (SD = 3.32) years for the HZ and non-HZ cohorts, respectively. As shown in Fig. 1, the cumulative incidence of PAD estimated using Kaplan–Meier analysis was significantly different among the 2 cohorts over the follow-up period (*P* < 0.001). After adjustment for age, sex, and comorbidities, the incidence density rates of PAD were higher in the HZ cohort than in the non-HZ cohort (4.64 vs 3.81 per 1000 person-years), with an adjusted HR (aHR) of 1.13 (95% CI 1.09–1.16) (Table 2). In both cohorts, the sex-specific relative risk of PAD was significant for women (aHR 1.16, 95% CI 1.11–1.22) and men (aHR 1.08, 95% CI 1.03–1.13). Furthermore, in both cohorts, the age-specific relative risk of PAD was higher for all age groups (age ≤49 years: aHR 1.27, 95% CI 1.20–1.35; age 50–64 years: aHR 1.14, 95% CI 1.08–1.21; and age ≥65 years: aHR 1.09, 95% CI 1.03–1.15). Regardless of the presence of comorbidities, the patients with HZ had a higher risk of PAD than did those without HZ.

**Table 1:**
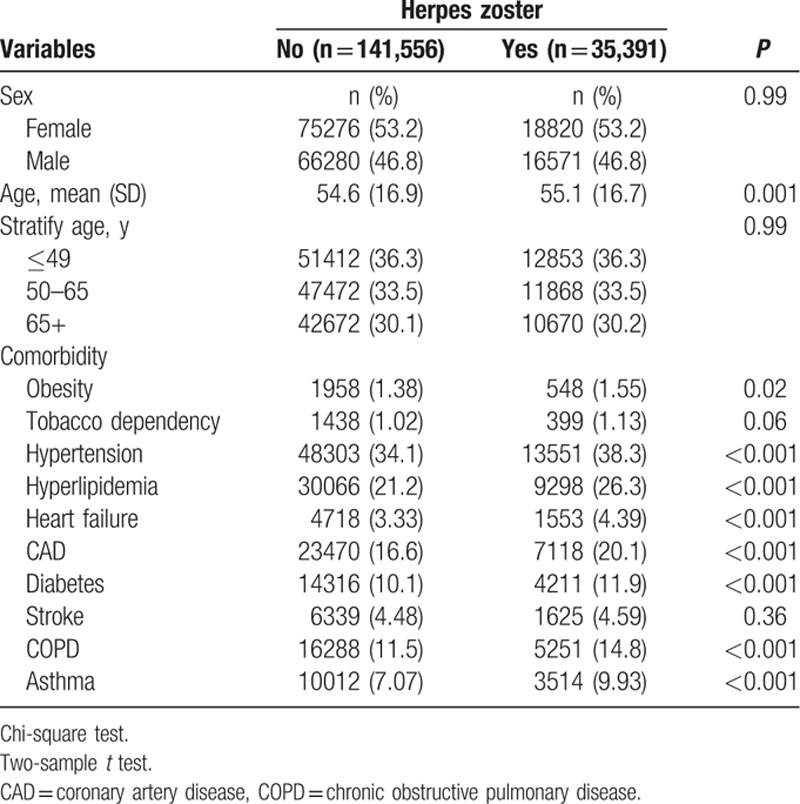
Demographic characteristics and comorbidity in patients with and without herpes zoster.

**Table 2:**
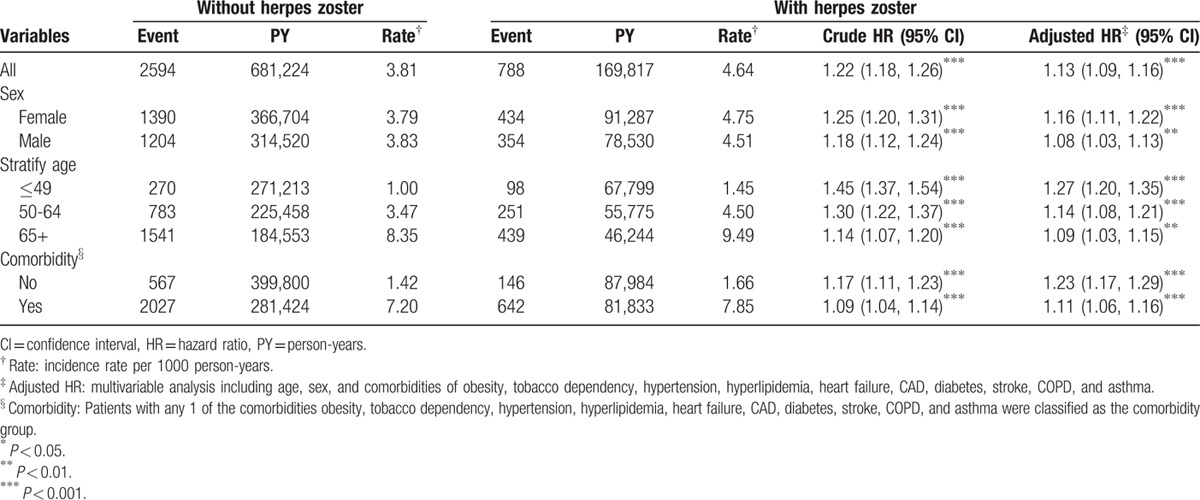
Comparison of incidence and hazard ratio of peripheral artery disease stratified by sex, age, and comorbidity between with and without herpes zoster patients.

The results of the univariable and multivariable Cox proportional-hazards regression models for analyzing the risk factors for PAD are shown in Table 3. Compared with the male patients, the female patients had a 3% increased aHR of PAD (aHR 1.03, 95% CI 1.00–1.06); this aHR increased 4% risk (aHR 1.04, 95% CI 1.03–1.04) with age (in 1-year intervals). The risk of PAD was higher in patients with comorbidities, namely diabetes (aHR 1.71, 95% CI 1.66–1.77), hypertension (aHR 1.62, 95% CI 1.56–1.68), tobacco dependency (aHR 1.32, 95% CI 1.13–1.55), hyperlipidemia (aHR 1.30, 95% CI 1.26–1.34), CAD (aHR 1.30, 95% CI 1.26–1.35), heart failure (aHR 1.14, 95% CI 1.08–1.20), and stroke (aHR 1.10, 95% CI 1.04–1.15). Moreover, the risk of PAD was not significantly higher in the patients with HZ who received the antiviral treatment than in those who did not receive the treatment (aHR 1.00, 95% CI 0.92–1.08) (Table 4).

**Table 3:**
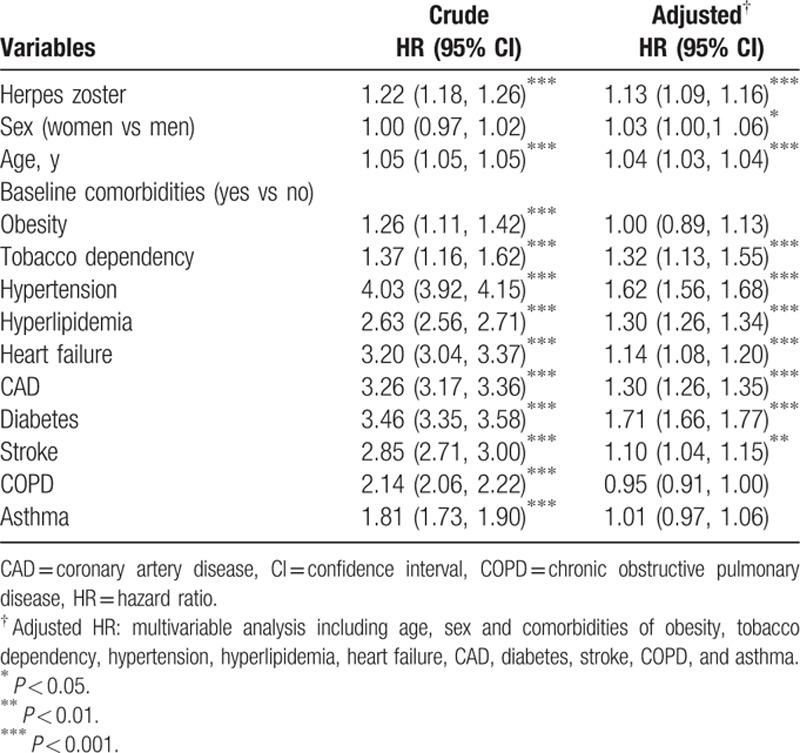
Hazard ratio of peripheral artery disease in association with sex, age, and comorbidities in univariable and multivariable Cox regression models.

**Table 4:**
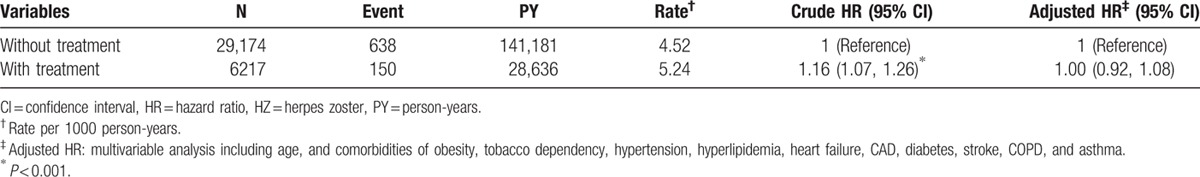
Comparisons of differences of incidence of peripheral artery disease within HZ cohort with or without antiviral treatment.

## 4 Discussion

This study is the first to elucidate the risk of PAD in patients with HZ by using a nationwide database. In this population-based cohort study, we adjusted several traditional risk factors for PAD and reported that HZ is an independent risk factor for PAD. In our study, the risk of PAD was 13% increased in the HZ cohort than in the non-HZ cohort.

Multiple mechanisms underlie the risk of PAD in patients with HZ. First, HZ infection may induce an increase in several prothrombotic autoantibodies. Uthman et al^[17]^ observed anticardiolipin antibodies of IgG and IgM in a patient with deep vein thrombosis complicated with zoster infection. Second, after reactivation from the ganglia, the infection transaxonally spreads to arteries and causes pathological vascular remodeling.^[18]^ HZ infection is characterized by a disrupted internal elastic lamina, intimal hyperplasia, and decreased smooth muscle cells in the medial layer.^[19]^ Similar morphological changes were reported in atherosclerosis, in which inflammation has emerged as a pathogenic factor.^[20]^ Similarities exist between the pathophysiological processes in patients with VZV and arterial wall processes during atherosclerosis progression, subsequently resulting in atherosclerotic plaque rupture and thrombotic vascular events. The findings of these basic experimental studies are consistent with those of our epidemiological study.

Most patients with HZ in this study were women, and PAD risks increased in both men and women with HZ. The age-stratified effect of HZ on PAD development was the highest in patients aged <49 years. However, the incidence of PAD increased with age in both the patients with and without HZ infection, which is consistent with a previous study.^[21]^ Lin et al^[22]^ reported that frailty, which is commonly associated with aging, can cause peripheral vascular disease. In our study, patients with comorbidities had an increased risk of PAD compared with those without HZ (aHR 1.11). Our study revealed that in addition to well-known risk factors such as hypertension, diabetes, and hyperlipidemia, HZ is also a risk factor for PAD.

This study could not determine whether the antiviral treatment for HZ infection successfully reduced the risk of PAD. However, this result has several potential confounding factors. First, the NHI reimburses the cost of antiviral therapy only for immunocompromised patients and those with complications. The patients who received the antiviral treatment may have had more comorbidities and traditional risk factors for PAD than did the patients who did not receive the treatment. Second, the NHI reimburses expenses for only 5 to 10 days of antiviral treatment. Third, the treatment initiation time and patient compliance were unavailable in the National Health Insurance Research Database (NHIRD). Additional prospective randomized controlled studies are necessary to determine whether antiviral treatment effectively reduces the risk of PAD after HZ infection.

The findings of this study need to be interpreted in the context of the following limitations. First, the insurance data do not contain detailed information regarding family history of PAD, current use of medications such as hormone replacement therapy, or previous anticoagulant treatment, which might have influenced the primary outcomes. Second, some patients with HZ might have preferred to not seek medical help. Third, our study had some asymptomatic patients with PAD, which may have resulted in the underestimation of PAD in the HZ and non-HZ cohorts. Fourth, evidence derived from a retrospective cohort study is generally of lower statistical quality than that from randomized trials because of potential biases associated with adjustments for confounding variables.

In conclusion, this study examined a nationwide population-based database containing a relatively high number of HZ cases and revealed that patients with HZ infection have a higher risk of PAD than do those without HZ. Physicians should carefully monitor vascular complications when treating patients with HZ.
